# Human transposon insertion profiling by sequencing (TIPseq) to map LINE-1 insertions in single cells

**DOI:** 10.1098/rstb.2019.0335

**Published:** 2020-02-10

**Authors:** Wilson McKerrow, Zuojian Tang, Jared P. Steranka, Lindsay M. Payer, Jef D. Boeke, David Keefe, David Fenyö, Kathleen H. Burns, Chunhong Liu

**Affiliations:** 1 Institute for Systems Genetics and Department of Biochemistry and Molecular Pharmacology, New York University School of Medicine, New York, USA; 2 Department of Pathology, Johns Hopkins University School of Medicine, 733N Broadway, Baltimore, MD 21205, USA; 3 Department of Obstetrics and Gynecology, New York University Langone School of Medicine, 462 First Avenue, New York, NY 10016, USA; 4 Department of Cell Biology, New York University Langone School of Medicine, 462 First Avenue, New York, NY 10016, USA; 5 McKusick-Nathans Institute of Genetic Medicine, Johns Hopkins University School of Medicine, 733N Broadway, Baltimore, MD 21205, USA; 6 High Throughput (HiT) Biology Center, Johns Hopkins University School of Medicine, 733N Broadway, Baltimore, MD 21205, USA; 7 Sidney Kimmel Comprehensive Cancer Center, Johns Hopkins University School of Medicine, 401N Broadway, Baltimore, MD 21231, USA

**Keywords:** mobile genetic element, retrotransposon, TIPseq, whole-genome amplification, somatic mosaicism, tumour heterogeneity

## Abstract

Long interspersed element-1 (LINE-1, L1) sequences, which comprise about 17% of human genome, are the product of one of the most active types of mobile DNAs in modern humans. LINE-1 insertion alleles can cause inherited and de novo genetic diseases, and LINE-1-encoded proteins are highly expressed in some cancers. Genome-wide LINE-1 mapping in single cells could be useful for defining somatic and germline retrotransposition rates, and for enabling studies to characterize tumour heterogeneity, relate insertions to transcriptional and epigenetic effects at the cellular level, or describe cellular phylogenies in development. Our laboratories have reported a genome-wide LINE-1 insertion site mapping method for bulk DNA, named transposon insertion profiling by sequencing (TIPseq). There have been significant barriers applying LINE-1 mapping to single cells, owing to the chimeric artefacts and features of repetitive sequences. Here, we optimize a modified TIPseq protocol and show its utility for LINE-1 mapping in single lymphoblastoid cells. Results from single-cell TIPseq experiments compare well to known LINE-1 insertions found by whole-genome sequencing and TIPseq on bulk DNA. Among the several approaches we tested, whole-genome amplification by multiple displacement amplification followed by restriction enzyme digestion, vectorette ligation and LINE-1-targeted PCR had the best assay performance.

This article is part of a discussion meeting issue ‘Crossroads between transposons and gene regulation’.

## 1. Introduction

A large proportion of the human genome is composed of interspersed repeat sequences, and a small subset of these are actively propagating as mobile genetic elements [1,2]. Long interspersed element-1 (LINE-1, L1) is one of the most active and abundant mobile DNAs in the human genome, and LINE-1 sequences comprise about 17% of the genome [1]. Most LINE-1 are old, fixed elements (i.e. homozygous insertion alleles in any individual human genome). However, a small subset of full-length LINE-1 insertions, members of the Ta subfamily of *Homo sapiens*-specific LINE-1 (L1Hs), are the evolutionarily youngest elements and have significant potential to retrotranspose through target primed reverse transcription (TPRT) [3–11]. These active LINE-1 are not only responsible for their retrotransposition, but also encode proteins that retrotranspose other repeat sequences in *trans*, namely, short interspersed elements (SINEs) and SVAs (SINE/VNTR/*Alu*) [12,13]. L1Hs elements, *Alu*Y SINE sequences and SVA insertions propagated by LINE-1 machinery together represent a significant source of structural variation in human populations [14–21].

There are at least 124 reported disease alleles caused by LINE-1-mediated retrotransposition events in the germline or early development [22–24]. Emerging data show that LINE-1 proteins are highly expressed in cancers and that somatic LINE-1 retrotransposition is commonplace in many cancers, indicating that LINE-1 expression and retrotransposition contribute to the genome instability in these malignancies [25–33]. LINE-1 insertions are frequent structural variants segregating in human populations, and many are not incorporated in the human reference genome assembly [34]. To understand genetic variation caused by these sequences and to find de novo insertions that distinguish cancer genomes from normal, many efforts have been made to profile LINE-1 insertions genome-wide using targeted or whole-genome sequencing [11,16,31,35–47]. Our laboratories contributed an approach we termed transposon insertion profiling by sequencing (TIPseq) [31,41,42,47]. This method is based on an insertion site-specific amplification, and covers the 3′ end of the L1Hs and downstream (3′), adjacent unique genomic DNA.

Genome-wide LINE-1 profiling in single cells has applications in many areas of research. As a marker of cellular lineage, it could be used to understand patterns of growth during development, somatic mosaicism in various tissues and clonal evolution in cancers. Several specific features of mobile element insertions make them useful as phylogenetic makers. First, they are directional, meaning that there is no ambiguity in distinguishing the pre-existing allele and the derivative allele. The ‘empty’ or pre-insertion allele is the antecedent allele, and the LINE-1 insertion allele arises later. Second, they are ‘homoplasy-free’, meaning that LINE-1 insertions are each unique [48–50]. In addition to its exact location, a LINE-1 insertion can be distinguished from another allele by its length, structure and target site duplication. Thus, finding the same insertion in two cells is strong evidence of a common origin or identity by division. We also have a lot to learn about retrotransposition, including tissue tropisms for the activity of specific source elements, whether LINE-1 activity is continual or episodic, and contributions of genotype and environment to retrotransposition activity. It seems that the activity of ‘hot’ LINE-1 loci is not constant throughout oncogenesis, but rather, apparently time-limited activities of different LINE-1 elements can cause new insertions in distinct tumour subclones [35,41]. Because of this, LINE-1 somatic insertions could be useful lineage markers in cancer heterogeneity and evolution. Somatically acquired LINE-1 insertions have been observed in neuronal cells, though other tissues and many non-malignant diseases have been less well studied [51–58].

There have been significant barriers to the development of LINE-1 mapping for single cells, owing to the prevalence of chimeric artefacts and the highly repetitive nature of LINE-1 sequences [56]. Whereas we previously have described TIPseq protocols requiring micrograms of genome DNA [47], here we demonstrate that TIPseq can be applied to whole-genome amplified DNA from little starting material—the genomic DNA content of a single cell. With data analysis using a modified version of TIPseqHunter2 software [31], the approach provides investigators with an economical and rigorous method for LINE-1 insertion site mapping in single cells.

## 2. Methods

### (a) Cell line

The GM12878 lymphoblastoid cell line, which is one of the European HapMap cell lines [59], was obtained from Coriell Institute for Medical Research. GM12878 cells were cultured in RPMI 1640 medium supplemented with 2 mM l-glutamine (Quality Biological, cat no. 112-025-101), 15% FBS (Corning, cat no. 35-010-CV), 100 units ml^−1^ of penicillin and 100 µg ml^−1^ of streptomycin (Thermo Fisher, cat no. 15140122).

### (b) Single-cell sorting

Single-cell suspensions of the cultured cells were washed with PBS, resuspended in the buffer (PBS + 1% BSA) and passed through 70 µm cell strainers (BD Pharmingen, cat no. 352235). Then the cells were stained with 1 mg ml^−1^ propidium iodide (PI) solution. Live single cells were sorted into PCR tubes. Doublet discrimination gates, including SSC-Height versus SSC-Width gate, FSC-Height versus FSC-Width gate, and PI gate, were used to ensure only one live cell was sorted per well (electronic supplementary material, figure S1). Cell sorting was performed in the Flow Cytometry and Cell Sorting Core Facility at Johns Hopkins Bloomberg School of Public Health.

### (c) Whole-genome amplification

Single-cell whole-genome amplification (WGA) was performed using multiple displacement amplification (MDA) or multiple annealing and looping-based amplification cycles (MALBAC) methods. MDA was performed using REPLI-g Single Cell Kit (QIAGEN, cat no. 150343). MALBAC was performed using MALBAC^®^ Single Cell WGA Kit (Yikon Genomics, cat no. YK001B). The sequences of L1 primer and MALBAC-L1 primer that were added during WGA are 5′-AGA TAT ACC TAA TGC TAG ATG ACA CA-3′ and 5′-GTG AGT GAT GGT TGA GGT CTT GTG GAG AGA TAT ACC TAA TGC TAG ATG ACA CA-3′, respectively.

### (d) Quality control

The quality of the WGA for the samples was evaluated using qPCR. Twelve pairs of primers were designed for qPCR to amplify regions downstream of the 3′ end of fixed L1Hs insertions on different chromosomes (electronic supplementary material, table S1). Primers were synthesized by Integrated DNA Technologies (IDT). A sample containing 100 sorted cells was used as a positive control. qPCR was performed using SsoAdvanced™ Universal SYBR^®^ Green Supermix (Bio-Rad, cat no. 1725271) and run on Bio-Rad MyIQ™ Single-Color Real-Time PCR Detection System. Fold change was calculated based on the Ct value and normalized with the positive control samples.

### (e) Vectorette PCR

Whole-genome amplified DNA samples were digested with *Ase*I, *Bsp*HI, *Bst*YI, *Hind*III, *Nco*I and *Pst*I (New England Biolabs). Alternatively, the whole-genome amplified DNA was end-repaired by 5′ phosphorylated and 3′ dA tailing using the NEBNext Ultra II End Repair/dA-Tailing Module (New England Biolabs, cat no. E7546S). A pair of vectorette oligonucleotides (synthesized by IDT) corresponding to each restriction enzyme or T tail were annealed to form vectorette adaptors with the sticky end created. See sequences reported in [47]. Then the digested or repaired amplified genomic DNA were ligated with the vectorette adaptors using T4 DNA ligase (New England Biolabs, M0202S) at 4°C overnight. After ligation, PCR was performed with the L1 primer (5′- AGA TAT ACC TAA TGC TAG ATG ACA CA -3′) and the Vectorette Primer (5′- CTC TCC CTT CTC GGA TCT TAA -3′) using ExTaq (Takara, cat no. RR006A) with a touchdown programme (95°C 5 min; 95°C 1 min, 72°C 1 min, 72°C 5 min, 5 cycles; 95°C 1 min, 68°C 1 min, 72°C 5 min, 5 cycles; 95°C 45 s, 64°C 1 min, 72°C 5 min, 15 cycles; 95°C 45 s, 60°C 1 min, 72°C 5 min, 15 cycles; 72°C 15 min; 4°C hold).

### (f) Next-generation sequencing

About 2 µg of the vectorette PCR products were sheared to fragments of around 300 bp. Sequencing libraries were prepared using the KAPA HTP DNA Library preparation Kit (Roche, cat no. KK8234). Libraries were sequenced on an Illumina HiSeq 4000 with paired-end 150 bp reads. Sequencing was performed in NYU Langone's Genome Technology Center.

### (g) Data analysis using TIPseqHunter2 pipeline

Reference and non-reference L1Hs insertions were identified using a modified version of the TIPseqHunter2 pipeline [31]. Reads are trimmed using Trimmomatic [60] and then aligned to both to the hg38 reference human genome and to the consensus L1Hs sequence using bowtie2 [61]. Regions of hg38 that are continuously covered by aligned reads are identified as potential L1 primer amplification sites. Regions that do not have any reads that align to both the L1Hs consensus and to hg38 are excluded. TIPseqHunter2 then uses five features to separate true L1Hs insertions from noise:

1. length of the amplified region (from putative L1Hs insertion to vectorette ligation site),
2. mean coverage across the amplified region,
3. mean number of alignment mismatches per read,
4. presence of an intact polyA tail,
5. number of split reads that align partly to the amplified region and partly to the L1Hs consensus sequence.

A support vector machine (SVM) model with radial basis kernel is used to separate true insertions from false positive insertions. Two hundred fixed L1Hs insertions make up the positive training set [31]. Potential amplification regions must have a read for which one end aligns discordantly to hg38 and the other end aligns to the L1Hs consensus sequence at the 5′ end of the L1 primer binding site to be considered a candidate insertion. Regions that do not have such a read make up the negative training set. The SVM model then returns a probability that a candidate insertion is a true insertion. Insertions in pericentric heterochromatin are filtered out. TIPseqHunter2 accuracy is measured by sensitivity (i.e. true-positive rate or recall), defined to be the fraction of true insertions that are given a probability over some threshold (in this paper, we use 0.9) and positive predictive value (PPV; i.e. precision), defined to be the fraction of potential insertions with probability exceeding the threshold that are true insertions. If TP is the number of true-positive calls, FP is the number of false-positive calls and FN is the number of false-negative calls then

$$\text{sensitivity}=\frac{\text{TP}}{\text{TP + FN}},$$

$$\text{PPV = }\frac{\text{TP}}{\text{TP + FP}}.$$

### (h) Validation of unknown insertions

Validation of the unknown insertions was done by spanning PCR and 3′ junction PCR. Spanning PCR is designed to amplify the entirety of an insertion, with primers flanking the insertion site. 3′ junction PCR is designed to amplify the 3′ of LINE-1 insertion and the downstream flanking sequence using L1 primer in the 3′ of LINE-1 and the other primer in unique flanking sequence.

## 3. Results

### (a) Single-cell sorting and whole-genome amplification

**Figure 1.**
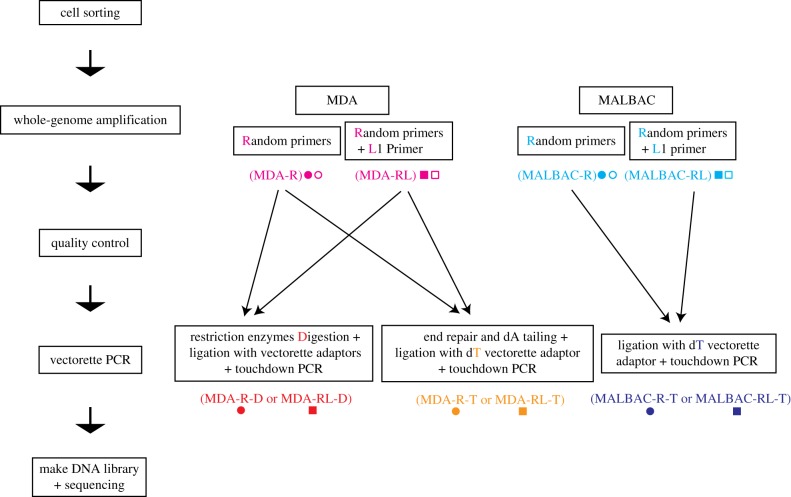
Overview of single-cell TIPseq workflows. The single-cell TIPseq procedure consists of five steps: cell sorting, WGA, a quality control check, vectorette PCR for L1Hs insertion site amplification and next-generation sequencing. Pink, MDA WGA with or without L1 primer (MDA); light blue, MALBAC WGA with or without L1 primer (MALBAC); red, MDA WGA followed by restriction enzyme digestion and ligation with vectorette adaptors (MDA-D); orange, MDA WGA followed by end repair, dA tailing and ligation with dT vectorette adaptor (MDA-T); dark blue, MALBAC WGA followed by ligation with dT vectorette adaptor (MALBAC-T). Circles represent WGA using random hexamers only (R); squares represent WGA using random hexamers and the L1 primer (RL).

The single-cell TIPseq procedure consists of five steps: cell sorting, WGA, a quality control check, vectorette PCR for L1Hs insertion site amplification and next-generation sequencing (figure 1).

Live GM12878 lymphoblastoid cells were sorted into PCR tubes or 96-well plates with one single cell per well. Multiple gates, including FSC-Height versus FSC-Width, SSC-Height versus SSC-Width, and PI, were used to make sure that only single live cells were sorted, and the dead cells and doublets were excluded (electronic supplementary material, figure S1).

Then single-cell genomic DNA was amplified by WGA. We tested two methods for WGA: MDA [62] and MALBAC [63]. For each method, we also tested whether amplification was improved by the addition of L1Hs-specific primers, which is called ‘L1 primer’. This L1 primer, ending with base pairs ‘ACA’, is designed to perfectly bind elements in the Ta subfamily, which is the youngest and most active subfamily of L1Hs, and causes most de novo retrotransposition in humans [8–11].

In the MDA WGA method, we tried the standard procedure with random hexamer primers in the reaction (MDA-R, ‘R’ to connote random primers); we also modified the procedure by adding additional L1 primer in the reaction (MDA-RL, ‘RL’ to connote random primers plus L1 primer). In both methods, the resulting amplicons ranged in size from 3 to 50 kb, and the yield was about 40 µg for MDA samples.

For the MALBAC WGA method, we tested the standard procedure (MALBAC-R), as well as the addition of the MALBAC-L1 primer in the pre-amplification step and L1 primer in the amplification step (MALBAC-RL). The size of amplicons ranged from 200 bp to 3 kb for MALBAC-R samples, and 100 bp to 3 kb for MALBAC-RL samples. The yield was 0.5–1 µg for MALBAC-R samples and 0.1–0.2 µg for MALBAC-RL samples.

### (b) Quality control of whole-genome amplification

Quality control of the single-cell WGA was performed by qPCR. We designed 12 pairs of qPCR primers in unique DNA sequence located at the regions 3′ of homozygous (fixed present) L1Hs insertions on 12 different chromosomes (electronic supplementary material, table S1). qPCR was performed using these 12 pairs of primers to evaluate the performance of the WGA in these regions. Although results were quantitative, amplification of these regions was essentially binary, with either low Ct values and robust amplification or negligible amplification, and so data are shown as numbers of loci amplified here.

**Figure 2.**
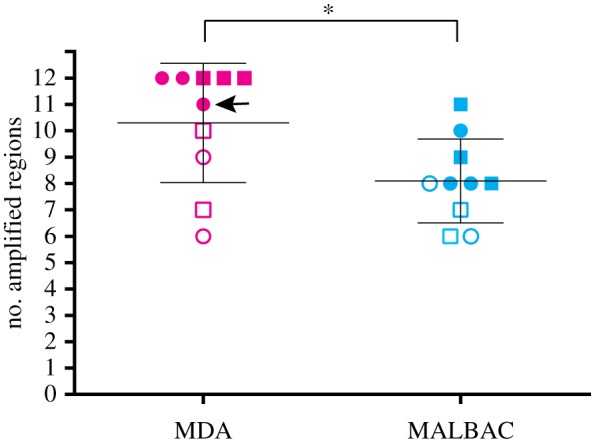
Quality control check following WGA. Pink, MDA WGA with or without L1 primer (MDA); light blue, MALBAC WGA with or without L1 primer (MALBAC). Circles, WGA using random hexamers only (R); squares, WGA using random hexamers and L1 primer (RL). Filled shapes, samples picked for following TIPseq; open shapes, samples that were not selected for the following TIPseq. An arrows indicates an MDA sample that is included in the following vectorette PCR and next-generation sequencing, but has only 11/12 regions amplified by qPCR, while other MDA samples have 12/12 regions amplified. *t-test, *p* = 0.0279.

A sample containing 100 sorted cells was used as a positive control, which showed 12/12 loci amplified. In a representative experiment (figure 2), 2 of 5 MDA-R and 3 of 5 MDA-RL samples showed effective amplification of all 12 regions (12/12). None of MALBAC-R or MALBAC-RL samples had uniform amplification of all 12 tested regions. Overall, MDA amplifies more regions of interest than MALBAC (*t*-test *p*-value 0.0279). These results suggested that MDA-based WGA had superior yield when compared with MALBAC-based WGA.

We picked three high-performing cell samples from each condition (MDA-R, MDA-RL, MALBAC-R or MALBAC-RL) for the subsequent vectorette PCR and next-generation sequencing in order to compare their performance. In the selected MDA samples, 5 out of 6 amplified all 12 regions, and the remaining 1 amplified 11 regions. In the selected MALBAC samples, none showed good recovery at all 12 loci.

### (c) L1Hs insertion site amplification and next-generation sequencing

After WGA and quality control, vectorette PCR was performed in those samples with consistently high qPCR amplicon yields. Vectorette PCR is a one-sided, ligation-mediated amplification reaction. For the MDA-R and MDA-RL samples, we tried two different template preparation procedures in advance of the amplification itself.

One preparation consisted of digestion of the amplified genomic DNA by restriction enzymes; ligation of the digested DNA fragments to the vectorette adapters that match the sticky end of the restriction enzymes; and touchdown PCR using the ligated products as the template, and using the L1 primer as forward primer and the vectorette primer as reverse primer. This is analogous to our usual vectorette PCR template preparation from bulk DNA samples. We term these reactions as MDA-R-D or MDA-RL-D, adding the ‘D’ to connote restriction digest.

The alternative procedure consisted of repairing the ends of whole-genome amplified genomic DNA to add 5′ phosphorylation and 3′ single nucleotide dA tails using a polymerase lacking 5'–3′ proofreading activity, then ligating the resulting DNA fragments to the vectorette adapter with a complementary dT overhang, followed by the touchdown PCR as above. These samples are called MDA-R-T or MDA-RL-T, the ‘T’ to connote tailing.

For the MALBAC-R and MALBAC-RL samples, because the last amplification step of MALBAC method adds a dA-tail to the 3′ end of the amplified fragments by *Taq* polymerase, and the sizes of the amplified fragments were only around 100 bp–3 kb, we skipped the digestion or repair steps, and directly proceeded to the single ‘sticky’ base A/T-mediated ligation of amplified fragments to the vectorette adapter. This was then followed by the touchdown PCR, the same as described above. These samples are referred to here as MALBAC-R-T and MALBAC-RL-T.

Then, for all samples, the vectorette PCR-amplified DNA was sheared to fragments at the size of around 300 bp. Then, DNA sequencing libraries were prepared and sequenced on an Illumina HiSeq 4000 with paired-end 150 bp reads.

### (d) Single-cell TIPseq results agree with known GM12878 L1Hs insertions

Before calculating the sensitivity, we developed a list of known L1Hs insertions in GM12878 cell line as ‘gold standard’ as described here. We began with an encompassing list of known L1Hs insertions, obtained by combining reference L1Hs loci with polymorphic insertions known to be present in GM12878 [20]. Then we applied two exclusionary criteria. First, since our L1 primer (ending with ‘ACA’) provides some specificity for the Ta subfamily of L1Hs insertions, which are the most active and youngest L1Hs in the human genome, we excluded the pre-Ta subfamily of L1Hs insertions, which have ‘ACG’ for the corresponding sequence. Using the whole-genome sequencing dataset of GM12878 (SRR622457 sequenced by the 1000 genomes project) [64], we removed insertions from our list that do not have exact matches to the L1 primer binding region. Second, we required that at least one read pair from SRR622457 align with one mate within 500 bp of the 3′ junction and the other covering the primer binding region with no mismatches. This excludes variant reference L1Hs that are missing from GM12878 and any L1 with observed sequence divergences within the primer binding region. After these exclusions, we were left with a list of 468 ‘gold standard’ L1Hs insertions expected in GM12878: 373 reference loci, 14 homozygous non-reference loci and 81 heterozygous non-reference loci. The ‘gold standard’ list can be found in electronic supplementary material, table S2.

**Figure 3.**
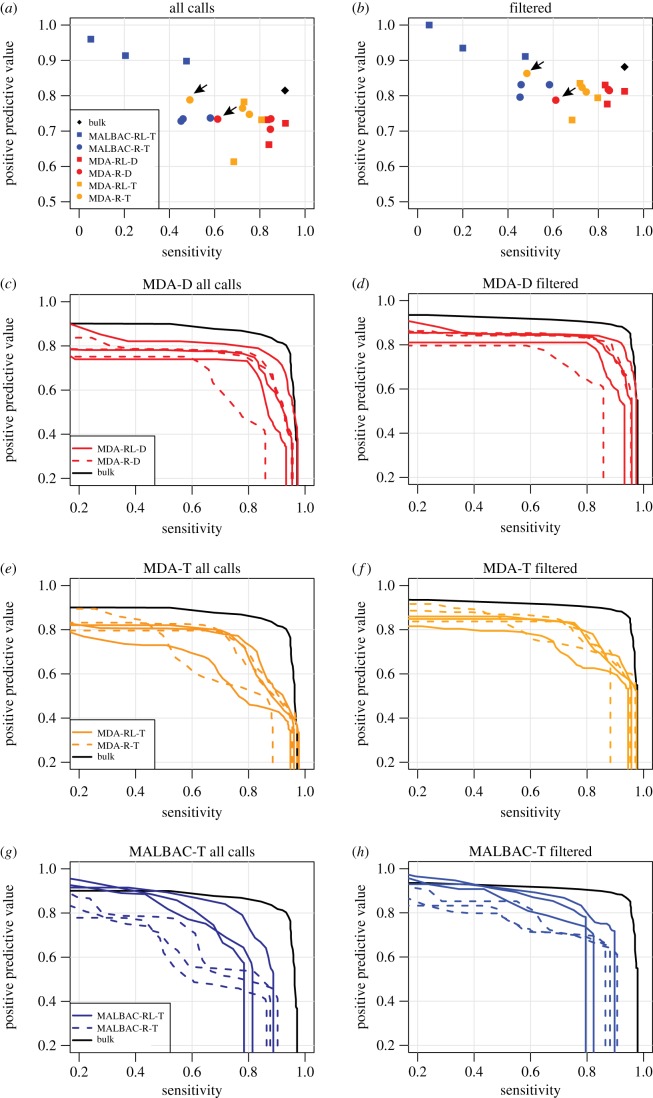
Comparison to ‘gold standard’ known insertions. Sensitivity and PPV when comparing single-cell TIPseq to a set of known GM12878 insertions with intact primer binding sites. (*a*) Sensitivity and PPV for all experiments, including all insertions and using a probability cut-off of 0.9. (*b*) As (*a*), but including only insertions that pass our three filters. Diamond, bulk DNA TIPseq; circle, WGA using random hexamers only (R); square, whole-genome amplification using random hexamers and L1 primer (RL). Arrows indicate the MDA sample included in the vectorette PCR (both MDA-D and MDA-T) and next-generation sequencing stages that had less than perfect QC (corresponds to the same sample indicated in figure 2). (*c–h*) Sensitivity–PPV curves for each single-cell TIPseq experiment as the probability cut-off is varied from 0 to 1. Black lines, bulk DNA TIPseq; red, MDA WGA followed by restriction enzyme digestion and ligation with vectorette adaptors (MDA-D); orange, MDA WGA followed by end repair, dA tailing and ligation with dT vectorette adaptor (MDA-T); dark blue, MALBAC WGA followed by ligation with dT vectorette adaptor (MALBAC-T).

TIPseqHunter2 pipeline was used to identify the L1Hs insertions in all the samples [31]. Insertions within 100 bp were merged as the exact location of an insertion can be hard to pinpoint, especially when L1Hs inserts into an A-rich region that blends with the polyA tail. Using a strict (svm probability > 0.9) cut-off and comparing to our gold standard list, we find single-cell TIPseq sensitivities (the fraction of gold standard insertions that are identified) as high as 90%, with 5/6 MDA-D experiments achieving sensitivity greater than 80% (figure 3*a*). Sensitivity is about 10% lower when only non-reference heterozygous insertions are considered (electronic supplementary material, figure S2). Thirteen of 18 single-cell experiments have PPVs (the fraction of insertions calls that are in the gold standard list) in the 70–80% range (figure 3*a*), with three samples exceeding 80% PPV and two falling below 70%. Because TIPseqHunter2 provides a probability score for each potential insertion, the cut-off can be made more or less stringent, improving either sensitivity or PPV at the expense of the other. The effect of this trade-off is shown in figure 3*c,e,g*. In our experiments, a strict cut-off (svm probability > 0.9) was used for TIPseqHunter2. The TIPseqHunter2 probability scores for each potential insertion in each experiment can be found in electronic supplementary material, table S2.

### (e) Unknown insertions filtering and validation

We next investigated those insertion calls made in an MDA-D experiment (our favoured protocol, see below), but that did not appear in our gold standard list. About one-third (*n* = 139) are real L1Hs that appear in euL1db [65], but were excluded from our gold standard list. These could be members of the pre-Ta subfamily of L1Hs in GM12878 that were non-specifically amplified (owing to only 1 bp mismatch with the L1 primer). Another sizeable fraction (*n* = 197) are in or within 25 bp of an L1PA2, L1PA3 or L1PA4 element. These calls are likely mis-amplification from L1 primer binding to highly similar sequences in evolutionarily older LINE-1 elements. Of the remaining 160 calls, 54 are in segmental duplications with greater than 95% similarity, and 79 are within 10 kb of known L1Hs element. The latter seem to reflect intramolecular rearrangements that result in chimeric MDA products [66], and ultimately false-positive calls proximal to true LINE-1 insertions. We incorporated more stringent criteria based on these observations, filtering out calls that are (i) within 25 bp of an L1PA2, L1PA3 or L1PA4 element, (ii) in a segmental duplication, or (iii) within 10 kb of a known L1Hs, and repeated our sensitivity and PPV analysis. These filters removed 34 gold standard L1Hs elements (30 reference, 4 heterozygous non-reference), while increasing PPV above 80% in 13/18 single-cell experiments (figure 3*b*). After applying this filtering across the five MDA-D samples passing quality control, on average 297 (80%) of the gold standard reference, 59 (73%) of the gold standard heterozygous non-reference and 12 (86%) of the gold standard homozygous non-reference insertions were detected. Across the 6 MDA-D experiments, 27 unknown predicted, but likely false, LINE-1 calls passed filtering. Only 2 of these 27 are predicted in bulk. Effects of this filtering strategy on PPV versus sensitivity plots are shown in figure 3*d,f,h*.

We next performed PCR validations on 4 of these 27 unknown insertions. We tried to amplify the LINE-1 insertion by spanning PCR with primers flanking the insertion sites. Owing to potential difficulty amplifying large LINE-1 of unknown size, we also attempted 3′ junction PCRs pairing the L1 primer in the 3′ of LINE-1 with a primer in unique, downstream flanking sequence. We recovered amplicons from bulk DNA and all whole-genome amplified, single-cell samples from the 3′ junction PCRs, but not the corresponding spanning PCRs. Sanger sequencing of the 3′ junction PCR products indicated that these are all non-specific PCR amplifications aligning to the wrong location of the genome. We also tested three candidates that would be filtered out as they are within 10 kb of known L1Hs, and we found all of them are artefacts caused by WGA. Consistent with our assumption that GM12878 is genomically stable with well-characterized LINE-1 variants, we did not identify novel LINE-1 insertions that could be validated by site-specific PCRs and Sanger sequencing.

Taken together, these findings show that TIPseq and TIPseqHunter data analysis provide near-complete profiles of LINE-1 insertion sites from single cells with infrequent false-positive calls after filtering. Subsequent manual curation and PCR validation of positive calls would still be needed to conclusively demonstrate retrotransposition in samples with somatic mosaicism.

### (f) Sensitivity for identifying L1Hs insertions is better in multiple displacement amplification samples than multiple annealing and looping-based amplification cycles samples

To compare the performance of single-cell TIPseq with WGA by MDA and MALBAC, we tested the sensitivity and PPV in detecting L1Hs insertions in both samples. When tested against our ‘gold standard’ GM12878 insertion list, MALBAC samples had poor sensitivities: average MALBAC sensitivity was only 37% compared to 75% for MDA (*t*-test *p*-value 0.004), although MALBAC did have slightly higher PPV (88 versus 81%, *t*-test *p*-value 0.06).

### (g) Restriction digested (MDA-D) templates perform best in single-cell TIPseq

We used two approaches to prepare the template for vectorette PCR. We digested the amplified genomic DNA with restriction enzymes, then ligated the digested DNA fragments with the vectorette adapter that matches the ‘sticky ends’ of the restriction enzymes (MDA-D); or we converted the amplified genomic DNA to repaired DNA with 5′ phosphorylated and 3′ dA-tailed ends, then ligated the repaired DNA fragments to the vectorette adapter that has dT-tailed ends (MDA-T). For MALBAC WGA products, we did not have an experimental arm to evaluate restriction digestion. Ends of the amplified DNA fragments were used directly in vectorette adapter ligations (MALBAC-T), because MALBAC-amplified fragments had dA tails at the 3′ end, and the size range of amplified fragments is smaller than MDA products, 100 bp–3 kb.

**Figure 4.**
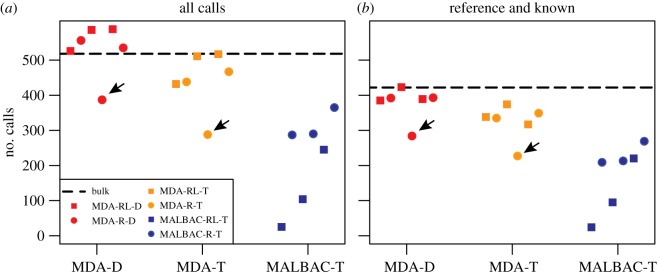
Total number of insertions predicted. (*a*) Including all predictions. (*b*) Including only known L1Hs insertion sites including the reference L1Hs and published polymorphic L1Hs. Black dashed line, bulk DNA regular TIPseq; red, MDA WGA followed by restriction enzyme digestion and ligation with vectorette adaptors (MDA-D); orange, MDA WGA followed by end repair, dA tailing and ligation with dT vectorette adaptor (MDA-T); dark blue, MALBAC WGA followed by ligation with dT vectorette adaptor (MALBAC-T). Circles, WGA using random hexamers only (R); squares, WGA using random hexamers and L1 primer (RL). Arrows indicate the MDA sample included in the vectorette PCR (both MDA-D and MDA-T) and next-generation sequencing stages that had less than perfect QC (corresponds to the same sample indicated in figure 2).

We found that TIPseqHunter2 called more L1Hs insertions in MDA-D samples when compared with MDA-T and MALBAC-T samples (figure 4*a,b*) (electronic supplementary material, table S2). When compared with our ‘gold standard’ GM12878, L1Hs insertion set excluding insertions in or within 25 bp of an annotated non-L1Hs reference L1, MDA-D samples (average sensitivity 82%) performed nearly on a par with a bulk DNA sample (sensitivity 92%), slightly better than MDA-T samples (average sensitivity 70%, *t*-test *p*-value 0.08) and much better than MALBAC-T samples (average sensitivity 37%, *t*-test *p*-value 0.002). PPV was not significantly different between MDA-D and MDA-T (81% versus 81% *t*-test *p*-value 0.88) and was only slightly better for MALBAC-T (81% versus 88%, *t*-test *p*-value 0.06) (figure 3*a,b*). In summary, MDA-D samples performed the best with highest sensitivity.

Not surprisingly, the subset of MDA samples that failed to amplify all 12 regions in the QC step but were carried forward to the vectorette PCR stage anyway performed worst in terms of the sensitivity detecting L1Hs insertions in both MDA-D samples (sensitivity 61% compared to average 86% for the other 5 MDA-D samples) and MDA-T samples (sensitivity 48% compared to average 73% for the other 5 MDA-T samples) (figures 2, 3*a,b* and 4*a,b* arrow pointed). This suggests that samples able to amplify all 12 regions in the QC step could be considered as good quality WGA samples to move forward to the vectorette PCR stage. Thus, QC is a good indicator of the performance potential of an individual sample.

### (h) Adding L1Hs-specific primers at whole-genome amplification does not improve sensitivity

We added L1Hs-specific primers into the WGA reaction of both MDA and MALBAC methods to see if this would skew these amplifications towards L1Hs 3′ downstream regions and improve sensitivity. We were not concerned about any chimeric products between the L1Hs-specific primers and random gDNA fragments, because they should be effectively excluded by TIPseqHunter2.

The quality control test following the WGA step showed no significant difference between the regular MDA whole-genome amplification (MDA-R) samples and those with added L1 primer in the MDA whole-genome amplification (MDA-RL) as far as numbers of loci recovered (*t*-test *p*-value 0.700). Similarly, no difference was appreciated comparing the regular MALBAC whole-genome amplification (MALBAC-R) and preparations using the L1 primer in the MALBAC whole-genome amplification (MALBAC-RL) (*t*-test *p*-value 0.856) (figure 2).

After the complete protocol, we compared the two approaches by evaluating their identification of known GM12878 L1Hs insertions. We found no significant difference between how MDA performed with or without L1 primer (MDA-RL and MDA-R) in sensitivity (86% for MDA-RL-D, 77% for MDA-R-D, *p*-value 0.36; 73% for MDA-RL-T, 71% for MDA-R-T, *p*-value 0.86) or PPV (81% for MDA-RL-D, 81% for MDA-R-D, *p*-value 0.99; 79% for MDA-RL-T, 83% for MDA-R-T, *p*-value 0.27) (figure 3*c–f*). For MALBAC samples, it yields a sensitivity of 24% for MALBAC-RL-T versus 50% for MALBAC-R-T (*t*-test *p*-value 0.17) and PPV 95% for MALBAC-RL-T versus 82% for MALBAC-R-T (*p*-value 0.03) (figure 3*g,h*). The inclusion of L1 primer provided modest improvement of PPV for MALBAC-T samples, but not sensitivity.

## 4. Discussion

LINE-1 is known to retrotranspose in the germline [67], during development [68] and in many human cancers [25–33]. It is possible that increased occurrence of LINE-1 insertions will characterize diseases like Fanconi anaemia [69] or ageing in normal tissues [70]. Single-cell LINE-1 mapping is an emerging tool that can be used to explore somatic mosaicism in benign tissues and genetic heterogeneity in malignancies. Single-cell LINE-1 mapping has been used as a marker of mosaicism in the human brain [51–56,58], and other tissues and disease states may prove important to explore. Despite interest in this topic, there are significant technical challenges inherent in single-cell LINE-1 mapping that have posed a barrier to studies in the field.

Here, we report a new method for single-cell LINE-1 mapping in single cells sorted from a well-characterized HapMap lymphoblastoid cell line. After WGA from single cells, we perform QC by qPCR to decide which amplified well enough to go on the TIPseq protocols. We designed 12 pairs of primers targeting regions 100–800 bp away from the 3′ polyA tail of homozygous L1Hs insertions on 12 different chromosomes. Our findings demonstrate that the samples which yielded amplification for all 12 primer pairs in the QC step showed better overall performance for genome-wide L1Hs insertion site detection. This indicates that the QC step is key to choose well-amplified samples, reducing sequencing cost [53].

In our experiments, we compared MDA- and MALBAC-based WGAs. In the QC step, MDA showed more consistent recovery of genomic sequences downstream of L1Hs than MALBAC. In the complete analysis, MDA followed by TIPseq had higher sensitivity for detecting L1Hs insertions than MALBAC followed by TIPseq. There are several differences in these WGA products: (i) the sizes of MDA fragments are larger, 3–50 kb, while MALBAC produces smaller fragments, only 100 bp–3 kb; and (ii) the amount of DNA produced by MDA is about 40 µg starting from one cell, while the amount of DNA produced by MALBAC is only about 0.1–1 µg. Because of the small fragment sizes, we did not subject MALBAC fragments to restriction digests. Thus, the ligation to vectorette oligonucleotides following digestion depends on a single A/T overhang rather than longer, ‘sticky end’ ligations, a factor that could reduce the ligation efficiency and reduce the numbers of amplicon templates for the L1Hs-specific vectorette PCR. Consistent with this, we see poorer performance of A-tailed MDA products in vectorette PCRs (MDA-T) when compared with those that have been restriction digested before ‘sticky end’ ligation (MDA-D).

Another single-cell 3′ focused L1 sequencing, L1 insertion profiling (L1-IP) [16], has been reported [53]. It uses MDA for WGA, followed by a nested PCR using a primer specific to L1Hs (‘AC’, amplifying Ta and pre-Ta subfamilies) and degenerate primers [16]. Single-cell TIPseq is similar, but uses no nesting in its L1 amplification step; we use an L1 primer more specific to the Ta subfamily of L1Hs (ACA) [9] paired with a specific vectorette primer [42,47]. The latter requires additional steps to ligate sequences corresponding the vectorette primer to the DNA templates. TIPseq also breaks up these amplicons before sequencing, potentially resulting in reads more distributed downstream of L1 insertions. Both TIPseqHunter [31] and the computational analysis performed for single-cell L1-IP [53] rely on classifiers that use features of the LINE-1 polyA and its juxtaposition with unique genomic sequence. Whereas single-cell L1-IP analysis generates training data through an iterative process that uses the result of a previous iteration to train next iteration, we used a training set generated from fixed present insertions and amplified regions that lack evidence for the L1 primer binding region. Furthermore, we also used an svm model with radial basis kernel rather than logistic regression. This allows a nonlinear classification boundary that may perform better when one feature strongly suggests insertion, but another feature does not.

Importantly, this study represents one of the first reports of a single-cell transposon insertion site profiling protocol that compares different conditions and tests a well-studied genomically stable cell line. Testing on a cell line with known LINE-1 insertions allowed us to carefully investigate the basis for artefacts that lead to false-positive calls and filter them out. It allows us to provide a robust estimate of the accuracy of this method.

In the future, the specificity and sensitivity of single-cell LINE-1 mapping could be further improved by harnessing emerging single-molecule, long-read sequencing technologies. In the interim, though, it is clear that deep coverage of the 3′ end of L1Hs insertion sites is possible by coupling WGA with standard TIPseq protocols. This approach is an economical and robust one for resolving the occurrence of retrotransposition events in single human cells.

## Supplementary Material

Table S1. Primers for quality control qPCR

## Supplementary Material

Table S2

## Supplementary Material

Figure S1

## Supplementary Material

Figure S2

## Data accessibility

All the sequencing data are deposited at NCBI Sequence Read Archive (SRA) with BioProject accession number PRJNA547805. TIPseqHunter2 software is available on github at https://github.com/FenyoLab/TIPseqHunter. The analysed data supporting this article have been uploaded as part of the electronic supplementary material.
