# Supplementary figures and images for: Human transposon insertion profiling by sequencing (TIPseq) to map LINE-1 insertions in single cells
Source: Philos Trans R Soc Lond B Biol Sci. 2020 Feb 10;375(1795):20190335. doi: 10.1098/rstb.2019.0335 (PMC7061987; doi:10.1098/rstb.2019.0335)

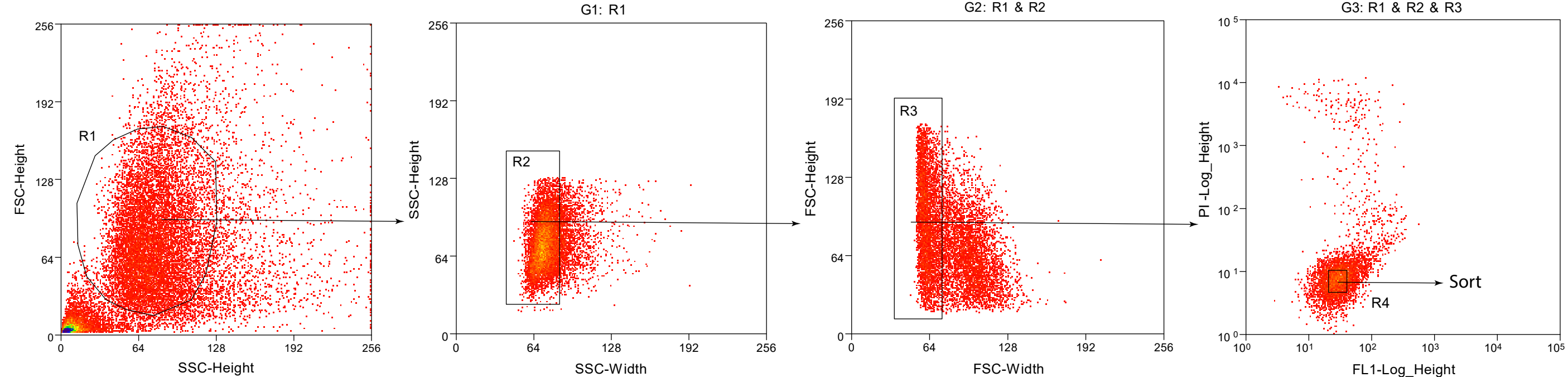

Supplement: Figure S1 [file rstb20190335supp3.pdf]

## Fraction of L1Hs identified

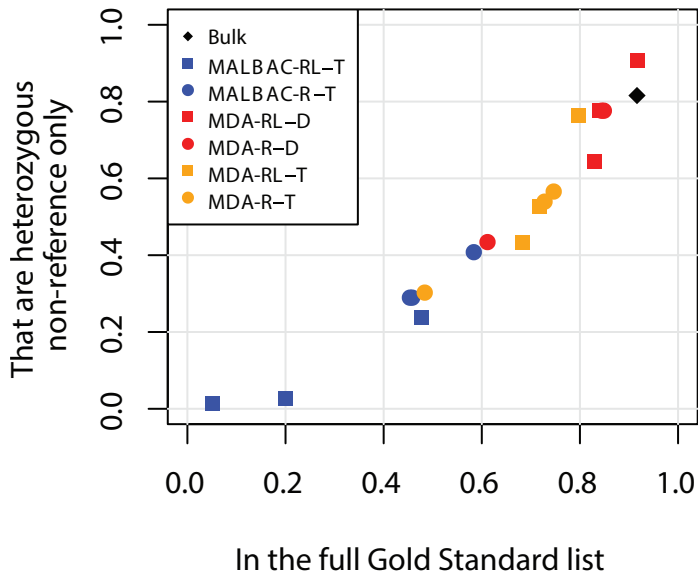

Supplement: Figure S2 [file rstb20190335supp4.pdf]
